# Thermosensing ability of TRPC5: current knowledge and unsettled questions

**DOI:** 10.1186/s12576-024-00942-3

**Published:** 2024-10-03

**Authors:** Alexandra Ptakova, Viktorie Vlachova

**Affiliations:** 1 Department of Cellular Neurophysiology, Institute of Physiology, Czech Academy of Sciences, Videnska 1083, 142 20 Prague 4, Czech Republic; 2 Department of Physiology, Faculty of Science, Charles University, Prague, Czech Republic

**Keywords:** Transient receptor potential canonical, Cold sensation, Voltage-dependent gating, Thermo-TRP channel, Stromal interaction molecule 1, Orai1

## Abstract

Our understanding of how the mammalian somatosensory system detects noxious cold is still limited. While the role of TRPM8 in signaling mild non-noxious coolness is reasonably understood, the molecular identity of channels transducing painful cold stimuli remains unresolved. TRPC5 was originally described to contribute to moderate cold responses of dorsal root ganglia neurons in vitro, but mice lacking TRPC5 exhibited no change in behavioral responses to cold temperature. The question of why a channel endowed with the ability to be activated by cooling contributes to the cold response only under certain conditions is currently being intensively studied. It seems increasingly likely that the physiological detection of cold temperatures involves multiple different channels and mechanisms that modulate the threshold and intensity of perception. In this review, we aim to outline how TRPC5 may contribute to these mechanisms and what molecular features are important for its role as a cold sensor.

## Background

Among the mammalian thermosensitive transient receptor potential (thermo-TRP) channels identified so far, three are activated by cooling or by noxious cold temperatures: TRPM8, TRPA1 and TRPC5 [1–3]. The last one identified was the canonical transient receptor potential 5 (TRPC5) [4], whose role as a transduction ion channel for cold is still the least explored. TRPC5 has been identified as a key molecular component for cold detection under certain neuropathic conditions [5] and for mediating cold pain in odontoblasts [6]. Its possible involvement in the regional adaptation to cold temperatures in the peripheral nervous system has been suggested [4]. TRPC5 knockout mice exhibit resistance to inflammatory pain and mechanical hypersensitivity, indicating that TRPC5 plays a role in the development of persistent pain after injury [5]. Since TRPC5 was first cloned [7–10] (then termed TRP5 or capacitative Ca^2+^ entry channel, CCE2), most functional studies have been performed in the indirect mode of its activation by G_q/11_ protein-coupled receptor stimulation, common to the entire canonical TRP (TRPC) receptor subfamily [11, 12]. The discovery of the specific TRPC4 and TRPC5 channel activator (−)-Englerin A [13] and the highly potent and selective inhibitor Pico145 [14] almost two decades later enabled more specific functional studies on TRPC5 in native and overexpression models. TRPC5 channels are found in various tissues, including the brain, but also in peripheral tissues, notably in the liver, heart, and kidney, gastrointestinal tract, blood vessels, ventricular myocytes and synoviocytes [12, 15]. An important role for TRPC5 in many physiological and pathophysiological processes has been described and includes the central nervous and cardiovascular systems, the kidneys and various metabolic disorders [12]. TRPC5 is also widely distributed in different cell populations of the human lung, suggesting the involvement of this channel in pulmonary cell function [16]. Recently, a missense variant in *TRPC5* has been identified (NM_012471.2:c.523C>T, p.(Arg175Cys)) in patients with an X-linked disorder presenting as intellectual disability and/or autism [17], and disruption of the *TRPC5* gene has been shown to cause obesity, behavioral problems such as anxiety, reduced social interactions, outbursts of aggression and, in mothers, postpartum depression [18]. A number of excellent reviews on various aspects of TRPC5 structure, pharmacology and physiological function have recently appeared, and we refer the reader to them [19–29]. Here, we review the progress in the understanding of molecular basis underlying polymodal gating of TRPC5 and focus on its thermosensitive properties.

### Structural features of TRPC5

**Fig. 1:**
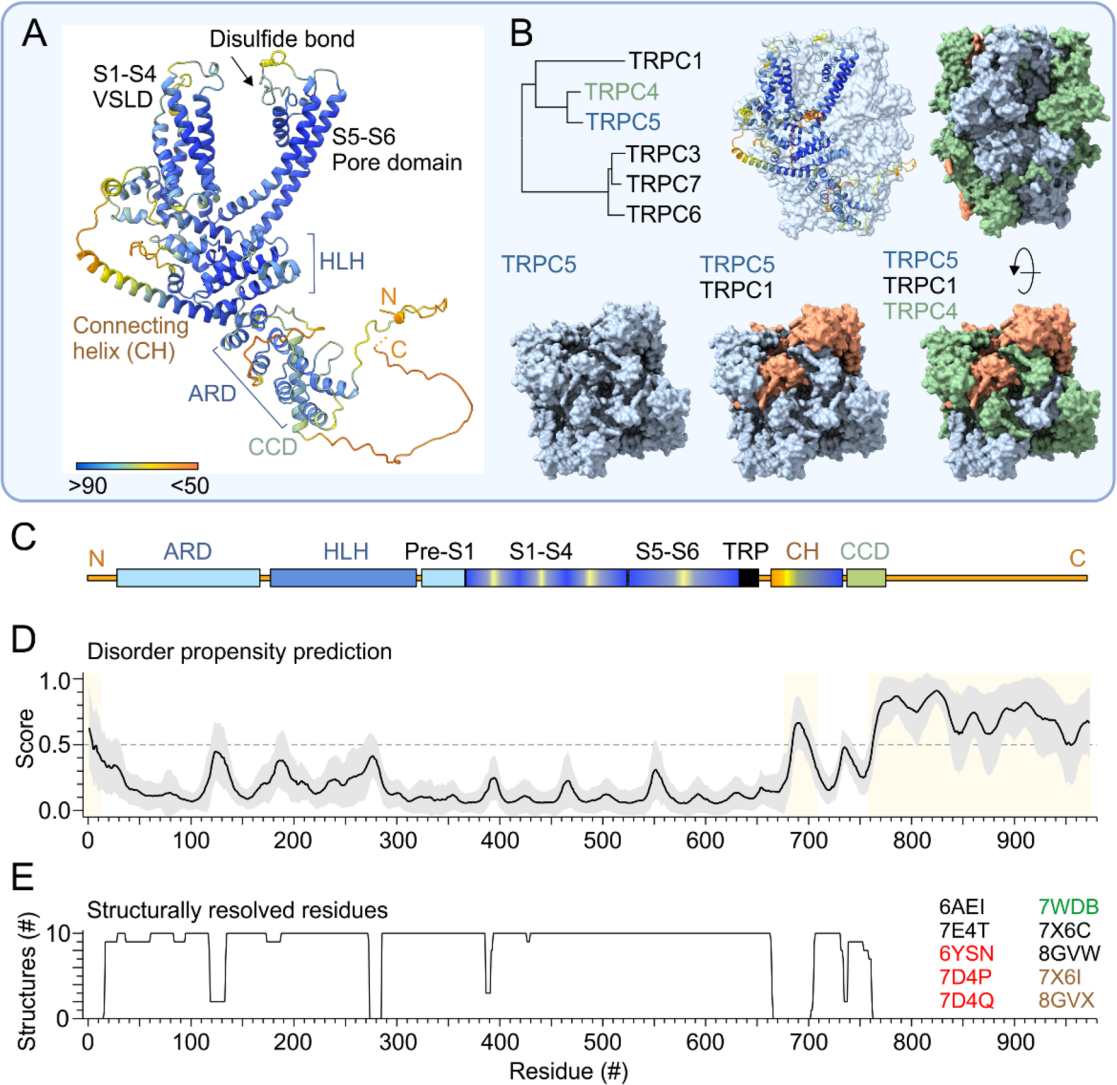
General architecture and intrinsically disordered regions of the TRPC5 channel. **A** Ribbon diagram of a single subunit of human TRPC5 generated using AlphaFold prediction server. The residues are colored using a per-residue confidence score pLDDT (predicted local distance difference test: scale bar with heat map from blue, pLDDT > 90; to orange, pLDDT < 50). Indicated domains: N-terminal akyrin repeat domain (ARD), helix-loop-helix domain (HLH), voltage sensor-like domain (VSLD) formed by the transmembrane spanning helices S1-S4, the pore domain formed by helices S5 and S6 and a reentrant loop in-between, containing a disulfide bond between cysteines Cys553 and Cys558 and a short pore helix. The C-terminus contains TRP helix (TRP), connecting helix (CH), coiled-coil domain (CCD) and a long (~ 200 amino acids) disordered region. **B** Phylogenetic tree generated for human TRPC1, TRPC3-7 amino acid sequences using MAFFT (v7) with default parameters. Surface map of the tetrameric structure of TRPC5 channel with indicated ribbon representation of one subunit (left) and as a heteromer with two TRPC4 subunits and one TRPC1 subunit. In native tissues, TRPC5 forms homomers or heteromers with TRPC1 and TRPC4. **C** Linear scheme of the TRPC5 domains shown in **A**. **D** Predicted average disorder score based on the sequence of human TRPC5 was obtained using PONDR VLXT, PONDR VL3, VLS2, IUPRED, PrDOS, AIUPred, AlphaFold, APOD, AUCPreD, DisEMBL, PreDisorder, DISOPRED3, IDP-Fusion, SPOT-Disorder and Metapredict. Line and gray envelope indicate mean ± standard deviation of the disorder score obtained from 15 prediction servers. Yellow shaded area (> 0.5) depicts disordered regions. **E** Structurally resolved residues in the 10 currently available structures of mouse or human TRPC5: The Protein Data Bank accession codes are indicated in black for apo-structures, in red for structures with inhibitors, in green for structure with activator riluzole, and brown for TRPC5 with the Gα_i3_ protein: 6AEI [32], 6YSN [36], 7E4T, 7D4P, 7D4Q [33], 7WDB [35], 7X6C, 7X6I, 8GVW, and 8GVX [34]

**Fig. 2:**
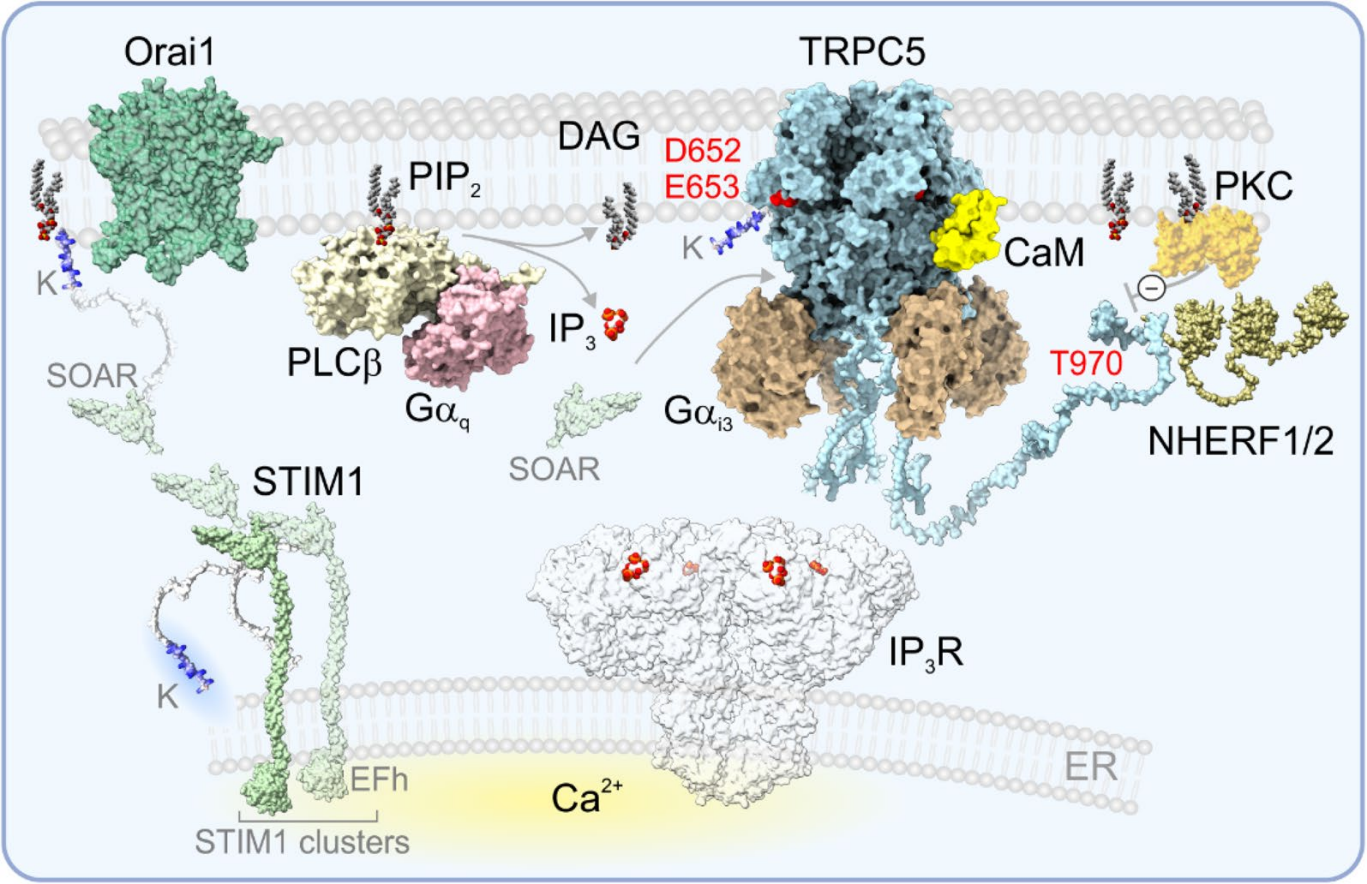
Schematic representation of main interactions of TRPC5 channel with signaling proteins in a cellular context. The indirect mode of TRPC5 activation is mediated downstream of the G_q/11_-protein coupled receptor activated phospholipase C (PLC) signaling pathway that leads to the cleavage of plasma membrane phosphatidylinositol 4,5-bisphosphate (PIP_2_) into two second messengers: diacylglycerol (DAG) and inositol 1,4,5-triphosphate (IP_3_), all of which are implicated in the regulation of TRPC channel activation. IP_3_ binds to IP_3_ receptors (IP_3_R), resulting in Ca^2+^ release from the endoplasmic reticulum (ER). The rise in intracellular Ca^2+^ concentration is sufficient and necessary to activate TRPC5. DAG activates the channel under specific conditions. A prerequisite for DAG sensitivity of TRPC5 is a dissociation of the scaffolding NHERF protein (Na^+^/H^+^ exchanger regulatory factor) from the PDZ-binding motif ”VTTRL” located right at the end of the C-terminus. This dissociation can be achieved in several ways: a phospho-null mutation of the former threonine from the “VTTRL” motif (T970), a charge-neutralizing mutation of the first PDZ domain of NHERF, inhibition of the protein kinase C (PKC), or the depletion of (PIP_2_). The PKC phosphorylation of T970 leads to TRPC5 desensitization, and PIP_2_ plays a role in maintaining PLC-independently evoked channel activity. Binding of Gα_i3_ subunit to the ankyrin repeat domain (IYY_57-59_ in the loop connecting ankyrin repeats 1 and 2) increases the sensitivity of TRPC5 to PIP_2_. In analogy with TRPC4, calmodulin (CaM) can interact with the connecting (rib) helix of the channel and restrict the mobility of the TRP helix, thus locking the channel in the closed state at high Ca^2+^ cytosolic concentrations. TRPC5 can partially function as a store-operated channel by interacting with the endoplasmic reticulum (ER) calcium sensor STIM1, the Stromal Interaction Molecule 1. After ER Ca^2+^ store depletion, STIM1 undergoes a conformational change and interacts with Orai1: STIM1 looses the Ca^2+^ bound to the N-terminal EF-hand domain (EFh) and undergoes from the tight state to the active, elongated state which allows it to interact with Orai1 (via the STIM1-Orai activating region; SOAR) and anionic phospholipids (via the lysine-rich region; K). This process is accompanied by oligomerization of STIM1 proteins to aggregates. Two positively charged lysines at the C-terminal end of STIM1 (in the K region) interact with two negative residues conserved among TRPC1/3/4/5/6 (D652 and E653 in TRPC5). TRPC is capable of interacting with SOAR and this interaction involves Y241, L244 and L255 in the H5 helix (N-terminal helix-loop-helix region). Importantly, the clustering of STIM1 at ER-plasma membrane junctions can be also induced by an increase in temperature above 35 °C, without depleting Ca^2+^ stores, and this process is highly temperature dependent. All structures shown are taken from the PDB database or were predicted by AlphaFold Protein Structure Database

As with other membrane proteins, it is only in the last few years that the molecular architecture of the TRPC5 channel and the precise mechanisms of its activation have begun to be better understood [30, 31]. Using advanced cryoelectron microscopy (cryo-EM) techniques, a total of 10 TRPC5 channel structures in near-atomic to atomic resolution have been published so far in different conformational states. The first cryo-EM structure of TRPC5 was resolved in 2019 and deposited in the Protein Data Bank under accession code 6AEI [32]. Other structures followed describing the apo state of the channel [33, 34] and its complexes with the activator riluzole [35], several inhibitors [33, 36] and Gα_i_ protein [34] (Fig. 1). The structure of TRPC5 consists of a transmembrane domain and a cytosolic domain comprising approximately 70% of the molecular weight of the channel. To form a functional ion channel, TRPC5 assembles into a tetramer with a fourfold symmetry consisting of either only TRPC5 subunits or can heteromerize with TRPC1 or TRPC4 subunits [37–39] (Fig. 1B). Each TRPC5 subunit consists of six transmembrane α-helices (S1-S6), with helices S1-S4 forming the voltage-sensor-like domain (VSLD) and the region between S5 and S6 forming the pore domain. The cytosolic region contains the N-terminal domain composed of four ankyrin repeats positioned below the helix-loop-helix domain (HLH, comprised of seven α-helices), and the C-terminal subdomain formed by a connecting helix (also termed „rib helix”) followed by a coiled-coil domain located beneath the permeation pore (Fig. 1A–C). The C-terminal segment including last ~ 210 amino acids (21.6% of the total sequence) represents an intrinsically disordered region (IDR) that is not resolved in any of the published structures (Fig. 1C–E). As in many other eukaryotic ion channels, the intrinsically disordered region is supposed to play important roles in channel function, localization and protein–protein interactions [40–43]. IDRs in proteins generally provide stability and resistance to cold treatment [44, 45], and so it is conceivable that the distal C terminus could provide proper TRPC5 channel function during cold-dependent activation. The distal C-terminal part contains the PDZ-binding motif „VTTRL” within which the first threonine T970 (human TRPC5 numbering) is a critical phosphorylation target for protein kinase C (PKC) [46]. Its phosphorylation promotes the association of the channel with Na^+^/H^+^ exchanger regulatory factors 1 and 2 (NHERF1/2), which in turn prevents the activation of the channel by diacylglycerol (DAG), a major physiological activator that controls the gating of most TRPC channel family members [47–49] (Fig. 2). The central ion conduction pathway has two major restriction points: the upper gate with the selectivity filter marked by glycine G581 and the lower gate formed by the side chains of I621, N625 and N629. TRPC5 is critically regulated by Ca^2+^ [50] that binds to the negatively charged intracellular pocket of the inner vestibule of VSLD [33]. This region is an important regulatory site and a potential target for various modulatory agents [33, 35, 51]. VSLD, near the S2-S3 and S4-S5 linkers, is occupied by a phospholipid, most likely phosphatidylinositol 4,5-bisphosphate (PIP_2_), which is critical for TRPC5 channel activation [34, 52].

### TRPC5 as a cold sensor in peripheral nervous system and in odontoblasts

TRPC5 is the only member among the TRPC family classified as a thermo-TRP channel. Its cold sensitivity was first discovered using electrophysiological and Ca^2+^-imaging recordings on HM1-HEK293 cells stably expressing human muscarinic type 1 receptor (hM1) and heterologously expressing the mouse TRPC5 orthologue [4]. In these experiments, TRPC5 activity was potentiated by temperatures decreasing from 37 to 25 °C, suggesting its possible role in mild cold temperature sensing. Series of behavioral assays including thermal place preference test and noxious cold, heat and mechanical stimulations were performed in mice (129S1/SvImJ strain) with deleted *TRPC5*. However, no shift in temperature-dependent behavior was detected when compared with wild-type mice. Despite these negative results, Zimmermann et al. [4] did not completely exclude the channel from being possibly involved in the mechanisms of thermosensation, especially in the range of 37–25 °C, but they concluded that TRPC5 is not essential for noxious cold sensing. The loss of TRPC5 led to significant adaptive changes. Skin-nerve recordings in intact animals revealed an increase in the number of fibers sensitive to menthol (a TRPM8 activator) and a lowered threshold for mechanical stimuli, indicating a potential functional replacement of TRPC5 with TRPM8 or other channels activated (or modulated) by menthol. In contrast, in isolated dorsal root ganglia (DRG) neurons, the TRPC5 deletion caused a decrease in TRPM8 expression and a reduction of cold-sensitive neurons. It has been suggested that cold sensitivity of TRPC5 could be crucial in different mechanisms of temperature adaptation, such as in modifying vascular perfusion, neurite outgrowth or in modulation of transcription or metabolism, rather than mediating a response to noxious stimuli.

It was not until a decade later that a significant involvement of TRPC5 in the detection of cold stimuli was discovered, in a mouse model of peripheral neuropathic pain [5] and in mouse teeth [6]. Using the spared nerve injury model of peripheral neuropathy, Sadler et al. demonstrated that global TRPC5 knockout mice failed to develop cold allodynia (measured as withdrawal latencies to hindpaw dry ice stimulation following spared nerve injury) in contrast to wild-type mice [5]. Bernal et al. studied the mechanisms of cold sensing in teeth, focusing on TRPC5, TRPM8 and TRPA1 channels [6]. TRPM8 together with TRPA1 are responsible for the majority of mild and noxious cold transduction in rodent skin [53] and multiple studies support their possible involvement in thermosensation in humans as well [54–58]. However, in dental temperature sensing, neither of these channels seem to play the crucial role [59]. Using a mouse model of evoked dental pulp injury, which has been associated with increased sucrose consumption [60], Bernal et al. [6] excluded both TRPM8 and TRPA1 from being essential in mechanisms of inflammatory tooth pain in mice, often accompanied by cold hypersensitivity [61]. On the other hand, a significant involvement of TRPC5 was discovered, as its genetic deletion in mice with evoked dental pulp injury resulted in no shift in behavioral response. Further experiments, focused on studying the cold-dependent mechanisms in the entire tooth sensory system, involving electrophysiological recordings from single nerve fibers in intact and healthy mice teeth, revealed an important involvement of TRPC5 but also of TRPA1 in cold transduction. This was further supported with pharmacological and genetic inhibition of these channels, which led to a significantly lowered cold responses and a reduction of cold-sensitive fibers. Ca^2+^-imaging of dissociated dental primary afferent neurons showed that the cold response was mediated mainly by TRPM8 and only slightly by TRPC5 and TRPA1. Quantification of TRPC5 protein revealed high expression in odontoblasts, specialized cells located at the outer layer of dental pulp creating a barrier between soft and hard tissue. Using TRPC5 reporter mice, the authors demonstrated that the expression of TRPC5 is largely restricted to the odontoblast cell layer in mouse and human teeth and suggested an essential sensory receptor function for TRPC5-expressing odontoblasts in tooth cold sensing [6].

### Cold-dependent properties of TRPC5 in heterologous expression system

The previously mentioned study by Zimmermann et al. [4] explored the thermosensing ability of TRPC5 in HM1-HEK293 cells stably expressing human muscarinic receptor type 1 (hM1) and transiently expressing mouse orthologue of the channel (C-terminally EGFP tagged). Whole-cell patch clamp recordings confirmed that TRPC5 is constitutively active at room temperature as described earlier [62] and revealed that lowering the temperature from 37 °C potentiates the channel. TRPC5 activity was described to be the most temperature sensitive in the range of 37–25 °C, which corresponds to a temperature coefficient *Q*_10_ ∼ 0.1, a value that is consistent with other thermo-sensitive (thermo-TRP) channels [63]. TRPC5 currents evoked upon cooling remained constant over membrane potentials ranging from − 40 to − 80 mV. The recorded cold responses were sensitized in the presence of carbachol (100 µM), an agonist of muscarinic receptors, previously conventionally used to indirectly activate TRPC5 channels via an activation of G_q/11_ protein-coupled receptors, and the currents were strongly enhanced after the addition of La^3+^. While lowering the temperature from 37 to 25 °C potentiated TRPC5 channel, subsequent warming to 40 °C decreased the TRPC5 mediated current responses, without and in the presence of carbachol and La^3+^. In native tissues, TRPC5 heteromerizes with TRPC1 forming an ion channel that is characterized by altered functional properties [37, 64]. When TRPC5 (C-terminally EGFP tagged) was co-expressed with TRPC1 (C-terminally YFP tagged), cooling from 37 to 25 °C did not stimulate heteromeric TRPC1/5 channels. The cold activation of TRPC5 was also demonstrated in this study using Ca^2+^-imaging recordings whereby an increase in intracellular calcium was observed in TRPC5 and hM1 expressing cells, however only after a carbachol application.

**Fig. 3:**
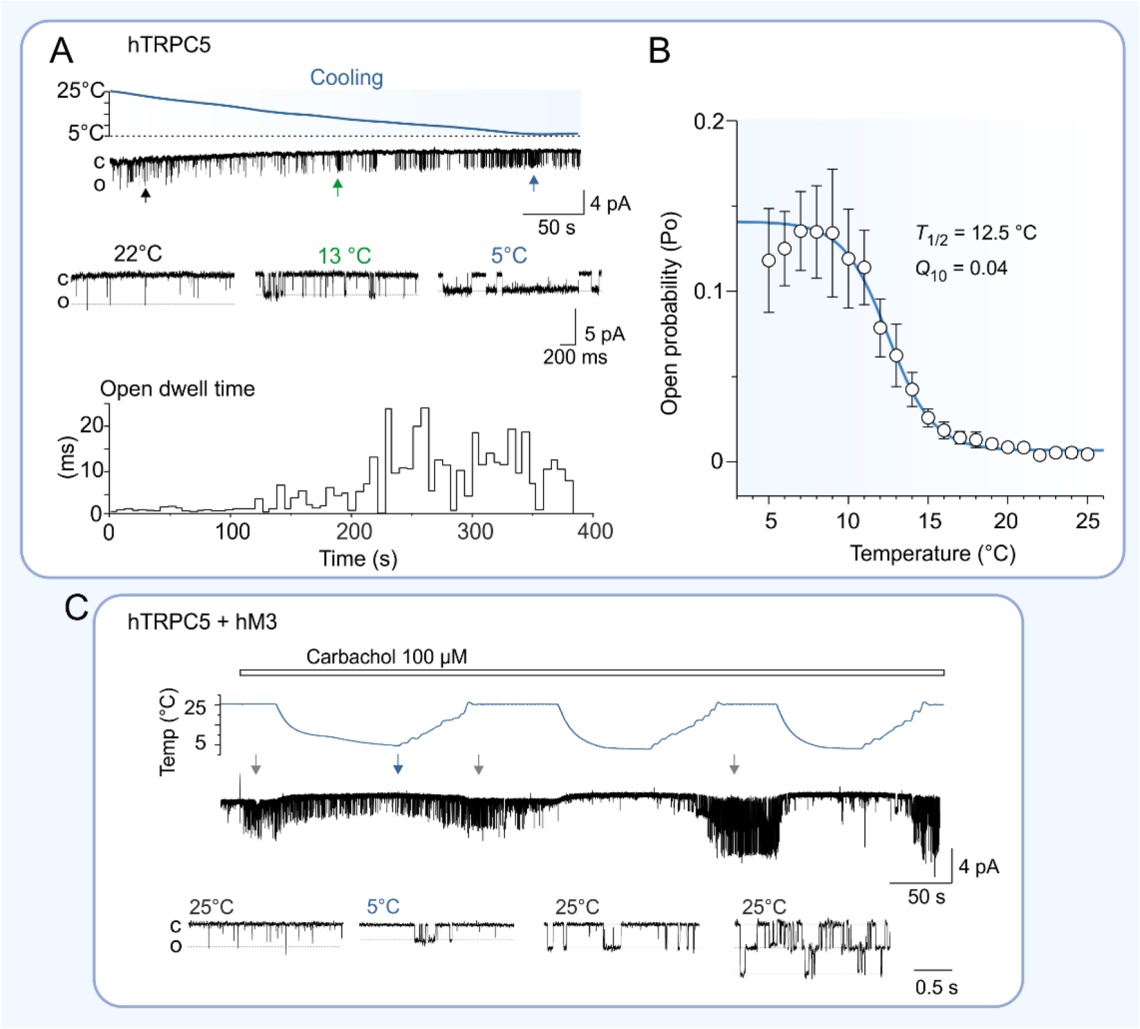
Changes in TRPC5 open probability upon cooling from 25 to 5 °C.** A** Representative recording of 7-min activity of one TRPC5 channel in response to cooling. 2 s expansions are taken at times indicated by colored vertical arrows. Single channel currents were measured from a HEK293T cell expressing human TRPC5 in cell-attached mode at pipette potential of + 120 mV. Downward deflections are inward currents; “o" indicates open level, “c” indicates closed level for each trace. Below, time course of mean open dwell times averaged over 6-s periods for recording shown above. Between 200 and 300 s (14–10 °C), the single-channel activity occurs in bursts separated by gaps. **B** Average open probability (Po) versus temperature plot shown as mean ± standard error of mean for 18 cells. The solid line is the best fit to a Boltzmann function. The estimated average temperature that causes 50% of the maximum response (*T*_1/2_) is 12.5 ± 0.01 °C. **C** Effects of temperature on TRPC5. The probability of TRPC5 opening is increased upon heating after cooling. Representative time course of unitary currents recorded in the presence of carbachol (100 µM) in cell-attached mode at + 120 mV from HEK293T cell co-expressing wild-type human TRPC5 with human muscarinic receptor M3. Below, 2-s expansions, taken at times indicated by colored arrows above. Note a strong increase in TRPC5 activity at 25 °C after the second and third cold stimulation. This figure was adapted from ref. [65]

The second published study exploring the temperature-dependent properties of TRPC5 activation is based on the single-channel cell-attached recordings carried out on intact HEK293T cells transiently expressing the untagged human TRPC5 orthologue [65] (Fig. 3A, B). At 25 °C, TRPC5 exhibited a basal activity manifested by short openings of the channel. Lowering the temperature to 5 °C prolonged the mean open dwell time and led to a strong increase in the open probability. TRPC5 gating was strongly temperature dependent (*Q*_10_ ∼ 0.04) between 16 and 11 °C. This was accompanied by changes in entropy and enthalpy, pointing to significant conformational changes. Around 8–5 °C, the channel activity became saturated. Cooling also affected the amplitude of unitary currents, which decreased ~ 1.5‐fold with a 10 °C drop in temperature. TRPC5 current responses to cold stimulation were further potentiated in the presence of the TRPC4/5 selective activator (–)-Englerin A [13] and were completely blocked by Pico145, a specific inhibitor of TRPC1/4/5 channels [14]. An effect of carbachol (100 µM) on single-channel activity was also tested on HEK293T cells co-expressing plasmids encoding TRPC5 channel and human muscarinic receptor type 3 (hM3). Carbachol application enhanced the cold-evoked current responses, however, it led to a reduction of temperature dependence of TRPC5 gating (*Q*_10_ ∼ 0.44). Milder cold dependence was also seen in the presence of (–)-Englerin A (*Q*_10_ ∼ 0.53). Analogous observations that the presence of an activator or sensitizer lowers the thermal activation threshold and temperature dependence have been seen for other temperature‐sensitive TRP channels [66, 67].

As noted above, threonine T970 in the distal C-terminus of TRPC5 (corresponding to T972 in murine TRPC5) is a critical phosphorylation target for PKC [46]. The T970A mutation disrupts phosphorylation by PKC, prevents desensitization that occurs after prolonged stimulation with carbachol [46, 52], and enables activation of the channel by DAG [49]. These processes are tightly controlled by PIP_2_ [52]. At a single-channel level, the T970A mutant exhibited increased basal activity at 25 °C and considerably lower temperature dependence than wild type channels (*Q*_10_∼0.22). The half-maximal temperature *T*_1/2_ was shifted by about + 4 °C, indicating an involvement of T970 in the cold activation mechanisms [65].

### Possible dependence of TRPC5 cold-induced activity on STIM1 and Orai1

The TRPC5 channel can directly interact with stromal interaction molecule 1, STIM1, and the extent of this interaction determines its function as a store-operated channel (SOC) [68–70]. STIM1 is a multidomain Ca^2+^ sensor with an amino-terminal EF hand Ca^2+^-binding domain residing in the endoplasmic reticulum (ER) (Fig. 2). In response to Ca^2+^ release from ER, STIM1 clusters at ER/plasma membrane junctions and activates two subtypes of Ca^2+^ channels in the plasma membrane, Orai and TRPC [71]. Whereas STIM1 is obligatory for the Orai channel activation [72], TRPC channels can function independently of STIM1 and Orai [73]. Exactly two STIM1 molecules are required to activate tetrameric TRPC channels [74] and the interaction of two conserved negative residues D652 and E653 with the polybasic K-domain at the C-terminal end of STIM1 is essential for TRPC5 activation [75].

The clustering of STIM1 is a highly temperature-dependent process [76, 77]. Heat induces STIM1 clustering that requires the polybasic K-domain and is independent of Ca^2+^ store depletion. Ptakova et al. investigated whether the cold-dependent activity of TRPC5 can be modulated by STIM1 [65]. They tested a “charge-swap” mutant of human TRPC5, in which D652 and E653 were replaced with lysines to prevent electrostatic interaction with STIM1. Compared with wild-type channels, the D652K/E653K mutant showed an increased probability of opening at 25 °C, which increased only slightly with decreasing temperature to ~ 8°C, and even decreased with cooling to 5 °C. The temperature dependence of unitary currents was no different from wild-type channels. These results indicate the involvement of STIM1 in cold-dependent TRPC5 activation. Next, the authors explored the possible role of endogenous STIM1 in TRPC5 activation. Using two different inhibitors of the sarco/endoplasmic reticulum Ca^2+^-ATPase pump to deplete intracellular Ca^2+^ stores, they did not observe any significant effects on the single-channel TRPC5 activity at 25 °C within 2 min of treatment, indicating that activation of endogenous STIM1 by store depletion is not determining the channel gating. They concluded that cold activation of TRPC5 depends on the electrostatic interaction of the channel with the polybasic K-domain of STIM1 but does not depend on intracellular Ca^2+^ stores. Overexpression of STIM1 caused both the wild-type TRPC5 and the double mutant D652K/E653K channels to become insensitive to cooling. The temperature of 37 °C is limiting for STIM1 protein that clusters at ER-plasma membrane junctions and, upon subsequent cooling to 25 °C, it activates endogenous Orai1 channels independently of Ca^2+^ store depletion [76]. TRPC5 is tightly regulated by intracellular Ca^2+^ [46, 78] and the overexpression of STIM1 might inhibit TRPC5 by disturbing Ca^2+^ homeostasis in transfected cells downstream of the endogenous store-operated channel Orai1. An interesting observation was that when TRPC5 was first exposed to cold, its activity increased strongly upon warming. This may be a physiologically relevant mechanism of TRPC5 regulation, as the probability of opening was also increased upon warming-after-cooling, when the channel was activated via a signaling pathway activated by the G_q/11_-protein coupled receptor M3 (Fig. 3C). Lee et al. [79] proposed a general model for the interaction between TRPC and STIM1: Under resting conditions, the N-terminus of TRPC interacts with the C-terminus and shields it from interaction with STIM1. Cell stimulation causes a dissociation of the interaction between the C- and N-termini, which enables STIM1 to bind and stabilize the open conformation of the channel. STIM1 interacts with TRPC via its STIM1 Orai1-activating region (SOAR) [74]. At low intracellular concentrations of Ca^2+^, SOAR is occluded by the other STIM1 domains and cannot interact with the channel (see [69], and references therein). The results presented in [65] may indicate that at low temperatures, the dissociation of the C- and N- termini of TRPC5 is largely delayed or prevented so that STIM1 cannot interact with and properly affect the channel.

STIM1 and Orai1 channels are present in peripheral neurons and a recent study by Buijs et al. revealed a new mechanism through which extreme cold (mean threshold around 10 °C) activates STIM1, causing aggregation of plasma-membrane Orai1 channels independently of Ca^2+^ store depletion [80]. This mechanism is prominent in sympathetic neurons and overlaps with another mechanism whereby cold suppresses two-pore potassium channels and triggers consequent membrane depolarization and action potentials via voltage-dependent calcium (Ca_V_) channels [81–84]. The authors demonstrated that the mechanism of cold transduction by STIM1 and Orai1 operates at a rather local level, without the generation and propagation of action potentials. Given that TRPC5 may co-occur with STIM1/Orai1 in certain cell domains (such as PIP_2_-rich domains as in the case of TRPC3 [85]), a similar concept could reconcile the current ambiguous observations regarding the role of this channel in cold detection.

### TRPC5 as a redox sensitive channel

One possible mechanism that may account for the cold sensitivity of TRPC5 is the cold stress-induced production of reactive oxygen species (ROS). It has previously been demonstrated that redox signaling initiated by mitochondrial ROS generation underlies the cold sensitivity of the TRPA1 channel [86]. Likewise, TRPC5 is a redox-sensitive channel that can be directly activated by both oxidants like hydrogen peroxide and antioxidants, including endogenous redox protein thioredoxin and chemical disulfide reducing agents dithiothreitol and membrane impermeable tris(2-carboxyethyl) phosphine hydrochloride (TCEP) [87]. The channel can also be modulated by reactive nitrogen species (RNS) such as nitric oxide via S-nitrosylation of cysteine residues C553 and C558 [88]. Recent structural and functional studies have confirmed that these two cysteines form a disulfide bond in the extracellular loop connecting S5 and the pore helix (Fig. 1A), and have demonstrated their key role in channel function, multimerization, trafficking and expression [32, 89–91]. The molecular mechanism through which cold-induced changes in ROS and RNS may affect TRPC5 gating likely involves destabilization of the upper region of the selectivity filter of the channel [91].

### TRPC5 as a potential drug target for cold pain

Currently, two TRPC5 inhibitors are registered in the ClinicalTrials database, GFB-887 targeting kidney disease and BI 1358894 targeting affective disorders (www.clinicaltrials.gov; NCT03970122, NCT04521478) [92, 93]. Both agents were found to be well tolerated by participants and have progressed to Phase 2 studies, but no TRPC5 inhibitor has yet been tested for cold or pain perception in humans. Although only an initial in vitro finding, the latest results suggest that TRPC5 may be a previously unsuspected target for duloxetine, a commonly used, highly effective drug for severe forms of pain [51]. Results from Ca^2+^-imaging, electrophysiology and molecular modelling demonstrated that duloxetine at clinically relevant concentrations inhibits human TRPC5 in a state-dependent manner, including a cold-dependent mode of activation, and its interaction site resides in an inner cavity of the VSLD. The inhibitory effect was observed in non-excitable HEK293T cells as well as in F11 cells derived from sensory dorsal root ganglia neurons. The authors hypothesized that duloxetine may contribute to analgesic effects in native peripheral neurons because it also inhibits the activity of homomeric TRPC4 and heteromeric TRPC1/TRPC5 channels. Duloxetine was originally introduced to treat major depressive disorder [94, 95] and later on has been repositioned to treat several painful conditions. Currently, it is the only drug that has successfully undergone clinical trials and demonstrated efficacy for severe cold-induced pain states associated with diabetic and chemotherapy-induced neuropathy [96, 97]. One of the peripheral mechanisms through which duloxetine exerts its antinociceptive effect is considered to be a blockage of voltage-gated sodium channels responsible for propagating action potentials [95, 98]. However, duloxetine is better at treating cold pain than other similarly acting drugs such as venlafaxine, thus the involvement of other targets can be anticipated [99]. Further testing of the effects of duloxetine in specific animal models could provide additional insights into the possible mechanism of action.

## Conclusions

In the last few years, new evidence has accumulated for the involvement of TRPC5 in cold perception, but a coherent picture has not yet emerged. Recently, the expression of TRPC5 in 75% of human sensory neurons has been demonstrated [5], however, the precise stoichiometry of TRPC5 channels in the peripheral nervous system is still unknown. Recent study by Kollewe et al. demonstrated that TRPC5 in rodent brain forms predominantly heteromers with TRPC1, TRPC4 and that only 9% of TRPC5 protein is present as homomers. Using high-resolution proteomics, the authors elegantly demonstrated that TRPC channels co-assemble with a number of interactors that provide specificity and efficiency of their function [38]. It is likely that analogous identification of TRPC5 interactomes in the peripheral nervous system will soon help elucidate the role of this channel in vivo. At this stage of the research, it seems safe to assume that the thermosensitive properties of TRPC5 are involved in the transduction of painful cold in the teeth [6], the detection of ambient cold with threshold at body temperature [4], and in the development of cold allodynia in a specific animal model of neuropathic pain [5]. Still, there are more questions than answers. Some of them can be directly formulated on the basis of the information resulting from this review article: How the STIM1-Orai1 cold transduction mechanism may affect TRPC5 in sensory and sympathetic neurons? Do neurons use TRPC5 to signal extreme cold, or does this channel function as an effector of the STIM1-Orai1 mechanism that operates at a local level without initiating a sensation propagating to consciousness? To what extent do different pro-algesic lipids, such as lysophosphatidic acid or lysophosphatidylcholine [100, 101], affect the temperature-dependent activation of TRPC5? Why is more significant activation of TRPC5 observed upon warming-after-cooling (i.e., upon removal of a cold stimulus) than when cold is held constant? Is cold-dependent activity of the C-terminally EGFP tagged mouse TRPC5 different from untagged human TRPC5? And how is the intrinsically disordered C-terminus of TRPC5 involved in cold-dependent gating of the channel? In a study by Buijs et al., the functional expression of TRPC5 in cultured DRG neurons correlated poorly with the sensitivity to a cold ramp from 32 °C to 4 °C [80]. The presence of TRPC5 was mainly tested by rosiglitazone (100 µM; a thiazolidinedione used as an antidiabetic drug). What would the functional profile of neurons look like if a more specific TRPC5 activator was used or the order of application of TRPM8, TRPA1 and TRPC5 agonists were reversed or combined with cold?

**Fig. 4:**
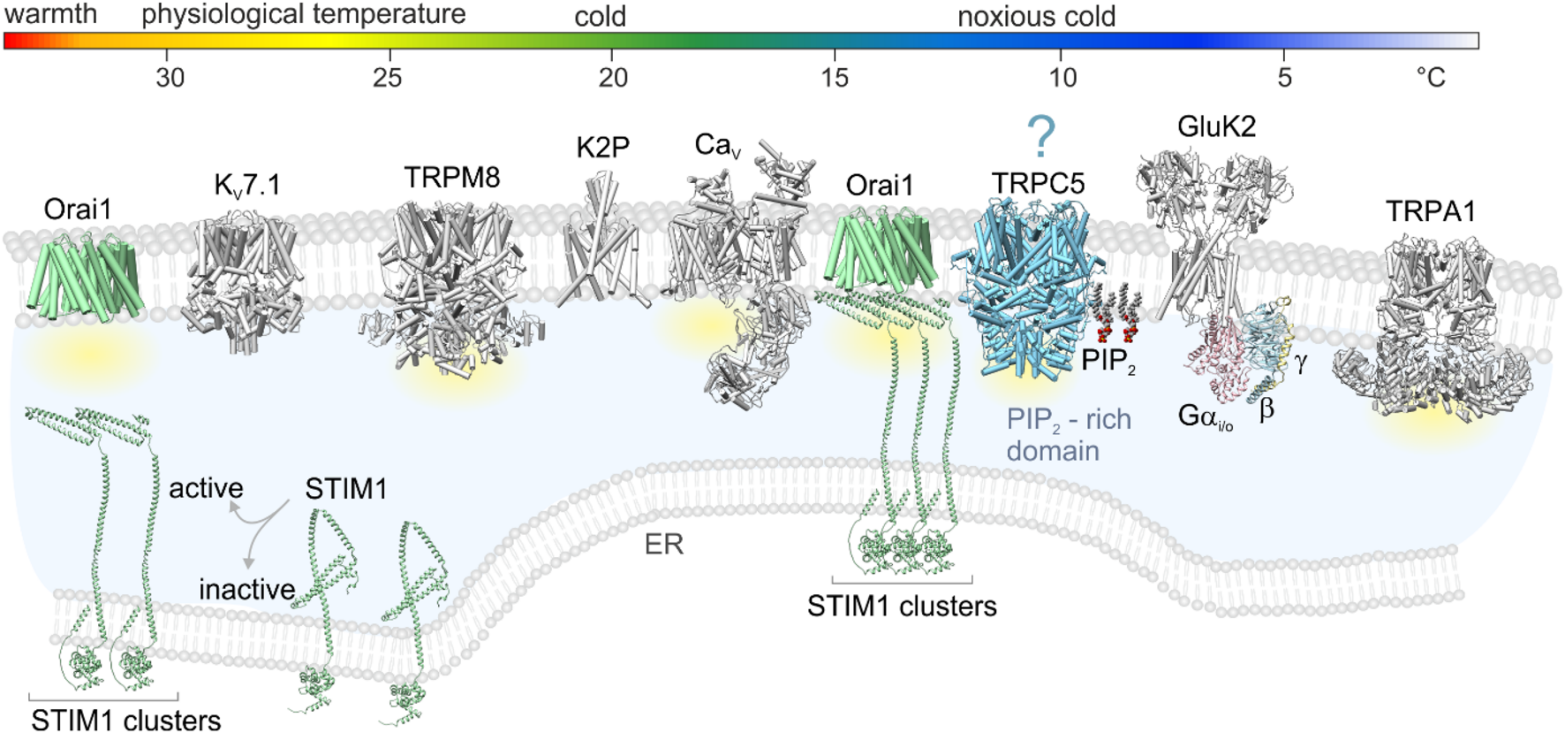
Summary model for cold transduction mechanisms activated by cooling in somatosensory and/or sympathetic neurons. TRPM8 channels are activated at mild cold temperatures below 23 °C. The Na^+^ and Ca^2+^ ions permeating through TRPM8 channels (yellow cloud indicates Ca^2+^ influx through the channels) lead to depolarization and subsequent activation of voltage-dependent calcium channels (Ca_V_) that can be blocked by verapamil. In the range of 25–35 °C, the voltage-gated potassium channels K_V_7.1 (KCNQ1) are inhibited and contribute to depolarization. The contribution of K_V_7.1 is sex-dependent. Stronger cold (threshold 13–15 °C) deactivates two-pore potassium channels (K2P), such as K_2P_10.1 (TREK2) and K_2P_18.1 (TRESK), causing depolarization, activation of Ca_V_ and calcium influx. Noxious cold < 10 °C induces translocation of STIM1 to regions adjacent to the plasma membrane, aggregation of Orai1 channels and their subsequent opening independently of intracellular Ca^2+^ stores. The clustering of STIM1 without depleting Ca^2+^ stores can be initiated also by heating cells above 35 °C, which leads to Orai1-mediated Ca^2+^-influx as a heat off-response. STIM1 is a dominant tether forming the endoplasmic reticulum-plasma membrane (ER/PM) junctions. Glutamate ionotropic kainate receptor 2 (GluK2), via its metabotropic function (G_i/o_-coupled), appears to be a major contributor to cold sensing in dorsal root ganglia neurons. Cooling may bring together, at the cellular and/or circuit levels, the channels in a PIP_2_ rich domain within the ER/PM junctions to enhance communication between the channels and enable their regulation by PIP_2_. The expression of some of these mechanisms may overlap. TRPC5 channels, which are highly sensitive to intracellular Ca^2+^ might work in concert with TRPA1 and STIM1-Orai1 mechanism to cover the range of noxious cold-sensation. The summary model was created based on the following articles: [2, 53, 65, 76, 80, 85, 102–104]

To stimulate further discussion and spark further research to decipher the exact mechanisms how TRPC5 contributes to cold transduction, we propose a hypothetical model for its possible role in the context of currently known mechanisms described in somatosensory and/or sympathetic neurons (Fig. 4).

## Data Availability

The datasets used during the current study are available from the corresponding author on reasonable request.

## Abbreviations

ARD: Ankyrin repeat domain
CaM: Calmodulin
Ca_V_: Voltage-dependent calcium channels
CCD: Coiled-coil domain
CCE: Capacitative Ca^2+^ entry
CH: Connecting helix
DAG: Diacylglycerol
DRG: Dorsal root ganglia
ER: Endoplasmic reticulum
HEK: Human embryonic kidney cells
HLH: Helix-loop-helix domain
hM1: Human muscarinic type 1 receptor
hM3: Human muscarinic type 3 receptor
IDR: Intrinsically disordered region
IP_3_: Inositol 1,4,5-triphosphate
IP_3_R: IP_3_ receptor
K2P: Two-pore potassium channels
NHERF: Na^+^/H^+^ exchanger regulatory factor
PIP_2_: Phosphatidylinositol 4,5-bisphosphate
PKC: Protein kinase C
PLC: Phospholipase C
PM: Plasma membrane
RNS: Reactive nitrogen species
ROS: Reactive oxygen species
SOAR: Stromal interaction molecule 1 Orai1-activating region
SOC: Store-operated Ca^2+^ influx channel
SOCE: Store-operated calcium entry
STIM1: Stromal interaction molecule 1
Thermo-TRP: Temperature sensitive TRP channel
TRPA1: Transient receptor potential ankyrin 1
TRPC5: Transient receptor potential canonical 5
TRPM8: Transient receptor potential melastatin 8
VSLD: Voltage-sensor-like domain
